# Anti-TROP2 Antibody Drug Conjugates in EGFR-Mutant Non-Small Cell Lung Cancer: Biological Rationale and Clinical Challenges

**DOI:** 10.3390/pharmaceutics18080905

**Published:** 2026-07-23

**Authors:** Laura Bonanno, Alberto Ronchi, Loc Carlo Bao, Francesca Pante, Sara Sangiorgi, Giulia Pasello, Stefano Indraccolo, Valentina Guarneri

**Affiliations:** 1 Department of Surgery, Oncology and Gastroenterology, University of Padova, 35128 Padova, Italy; alberto.ronchi@iov.veneto.it (A.R.); francesca.pante@iov.veneto.it (F.P.); sara.sangiorgi@iov.veneto.it (S.S.); stefano.indraccolo@unipd.it (S.I.); valentina.guarneri@unipd.it (V.G.); 2 Medical Oncology 2, Istituto Oncologico Veneto (IOV), IRCCS, 35128 Padova, Italy; 3 Basic and Translational Oncology Unit, Istituto Oncologico Veneto (IOV), IRCCS, 35128 Padova, Italy

**Keywords:** Osimertinib, resistance, EGFR, datopotamab deruxtecan, sacituzumab tirumotecan, interstitial lung disease, drug-tolerant persisters, ADC, TROP-2

## Abstract

The emergence of antibody-drug conjugates (ADCs) targeting trophoblast cell-surface antigen 2 (TROP2) represents a potential paradigm shift in the treatment of EGFR-mutated non-small cell lung cancer (NSCLC), a setting historically characterized by limited therapeutic options following progression on EGFR tyrosine kinase inhibitors (TKIs). This review examines the biological rationale and clinical challenges underpinning the development of anti-TROP2 ADCs in EGFR-mutated NSCLC. From a mechanistic standpoint, TROP2 occupies a unique and dynamic role in EGFR-mutated NSCLC, providing strong biological rationale for clinical development of anti-TROP2 ADCs in this population. Three TROP2-directed ADCs are currently in clinical development in this setting. Available clinical data in previously treated EGFR-mutated patients are critically reviewed here, focusing on efficacy, toxicity profiles, clinical challenges and potential future perspectives.

## 1. Introduction

The treatment landscape of epidermal growth factor receptor (EGFR)-mutant non-small cell lung cancer (NSCLC) has been revolutionized by tyrosine kinase inhibitors (TKIs). Despite unprecedented clinical responses, the emergence of acquired resistance mechanisms remains the primary obstacle to long-term disease control [1]. This unmet clinical need represents a compelling rationale for the exploration of novel therapeutic strategies. Efforts to improve outcome of EGFR-mutated NSCLC include the study of combination treatment upfront in order to delay acquired resistance mechanisms onset. In addition, new therapeutic strategies are under evaluation for patients experiencing progression following EGFR TKIs. In the latter subset, therapeutic strategies can be classified into two subgroups: targeted therapies according to the specific resistance mechanism detected in rebiopsy and new drugs studied in molecularly unselected patients [2]. Antibody-drug conjugates (ADCs) are a class of drugs designed to deliver potent cytotoxic payloads selectively to tumor cells and exploit the so-called bystander effect [3]. Among potential targets for ADCs, TROP2 has generated significant interest. TROP2 is a transmembrane glycoprotein frequently overexpressed in epithelial cancers, including NSCLC. Its expression is often associated with aggressive disease and poor prognosis in NSCLC [4,5]. Importantly, it has also been implicated as a potential resistance mechanism in EGFR-mutated NSCLC [6]. This biological rationale provides a compelling foundation for investigating anti-TROP2 ADCs in this clinical setting. However, the translation of this biological premise into clinical success for EGFR-mutated NSCLC still remains a challenge. This review examines the scientific basis for targeting TROP2 in this population and explores the key clinical challenges related to patient management and selection.

To maintain a clinically focused perspective, the scope of this review is intentionally restricted to the three TROP2-directed ADCs for which clinical data in EGFR-mutated NSCLC are currently available: datopotamab deruxtecan (Dato-dxd), sacituzumab tirumotecan (Sac-TMT), and sacituzumab govitecan (Sac-gov). While additional TROP2-directed ADCs are in earlier stages of development, none have reported clinical data in this molecularly defined population at the time of writing. This focus allows an in-depth analysis of the biological rationale, structure–activity relationships and clinical challenges.

## 2. Clinical Background and Biological Rationale

EGFR is a tyrosine kinase receptor that regulates epithelial cell proliferation and survival through the RAS/MAPK and PI3K/AKT cascades. Activating mutations—most commonly exon 19 deletions and L858R point mutations—promote ligand-independent signaling, leading to oncogenic addiction. Third-generation EGFR tyrosine kinase inhibitors (TKIs), such as Osimertinib, covalently bind the C797 residue to suppress mutant receptor activity and overcome T790M-mediated resistance. After being studied as an option for acquired resistant tumors, Osimertinib demonstrated to be superior to first- and second-generation TKIs in previously untreated patients [7].

While Osimertinib monotherapy has long been established as the standard first-line treatment for advanced EGFR-mutated NSCLC, combination strategies—namely Osimertinib plus chemotherapy and Amivantamab plus Lazertinib—have recently emerged as additional therapeutic options. Both combinations demonstrated superior efficacy compared to Osimertinib monotherapy in the overall study population; however, their adoption is tempered by an increased toxicity burden and the current absence of validated predictive biomarkers to guide patient selection [8,9].

Despite these advances, resistance to EGFR–TKIs is inevitable. Resistance mechanisms are broadly classified as primary (intrinsic) or secondary (acquired); the spectrum of acquired resistance mechanism differs according to the TKI administered as first-line treatment [7].

Primary resistance, defined by early disease progression (typically within the first six months) or lack of radiological response, may reflect incomplete EGFR dependency and is found in 4–10% of EGFR-mutant NSCLC patients treated with first line Osimertinib [7].

These mechanisms may result from structural heterogeneity of the receptor, such as exon 20 insertions that reduce inhibitor affinity, or from coexisting oncogenic alterations that maintain downstream signaling despite EGFR blockade. These include MET or HER2 amplification, PIK3CA and TP53 mutations, and PTEN loss and alterations in cell-cycle regulators such as CCND1/2 and CDK4/6. Furthermore, a germline BIM intron 2 deletion polymorphism can attenuate apoptosis and reduce TKI sensitivity [7,10].

Secondary resistance emerges after an initial period of clinical response and can be classified as “on-target” and “off-target”. On-target resistance arises from secondary EGFR mutations such as C797S, L718Q, or G796R, which hinder drug binding. Off-target resistance involves activation of alternative receptor tyrosine kinases (RTKs)—including MET amplification, HER2 alterations, or fusions involving RET, ALK, and FGFR—which reactivate the PI3K–AKT and RAS–MAPK cascades independently of EGFR. In a subset of patients, resistance manifests through histological transformation to small-cell lung cancer (SCLC), driven by TP53 and RB1 loss [7]. These mechanisms reflect a continuum of genomic evolution and adaptive signaling rewiring, where drug-tolerant persister (DTP) cells serve as a transient reservoir for resistance acquisition [7,10]. This adaptive plasticity underscores the need to investigate not only genomic drivers but also the cell-surface regulators that coordinate compensatory receptor signaling.

**Figure 1:**
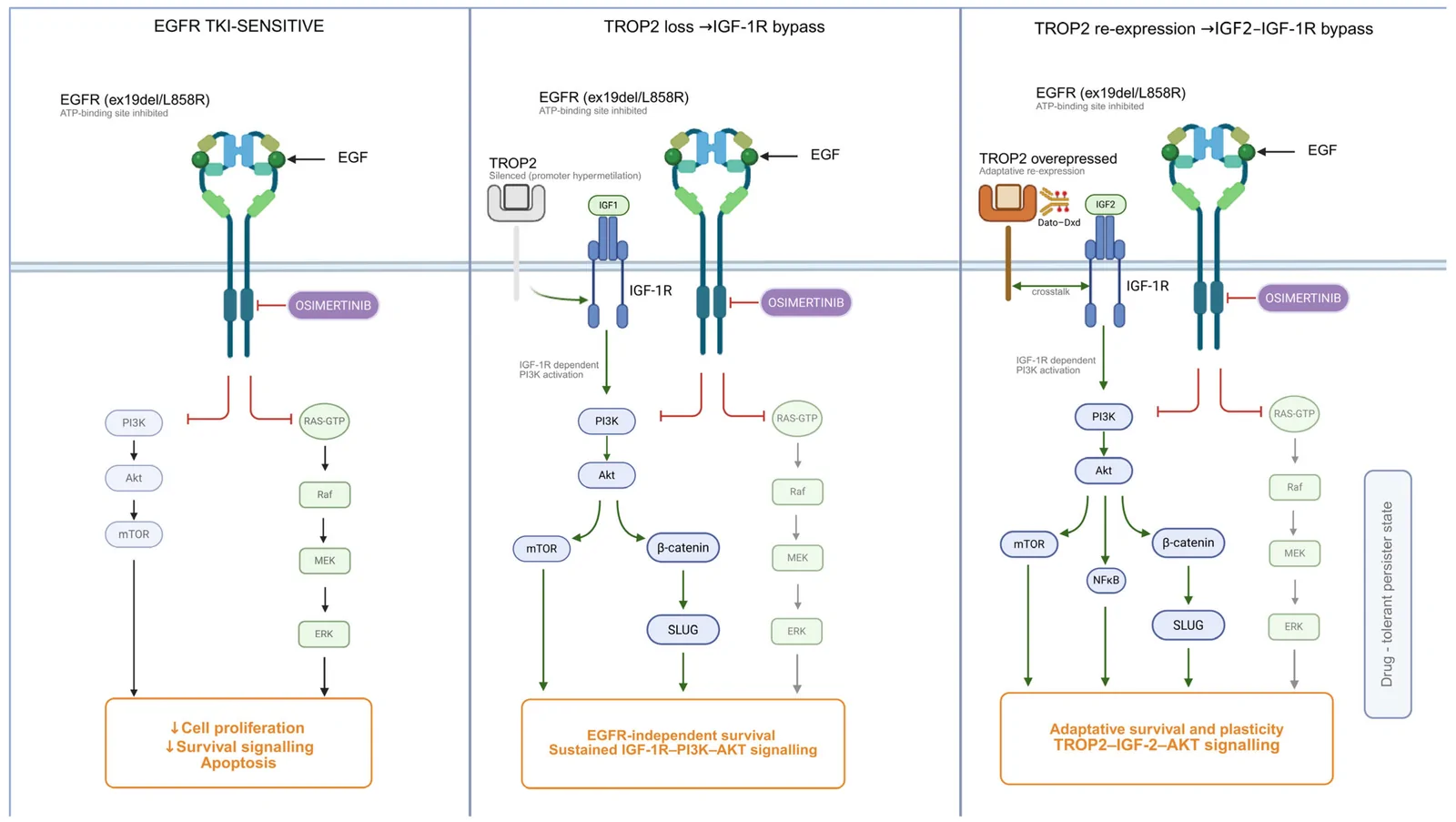
Dynamic role of TROP2 in EGFR-mutated NSCLC under osimertinib pressure. Created with BioRender (Ronchi, A. (2026), https://BioRender.com/2cu8rk0 (accessed on 4 April 2026), CC BY 4.0). Three sequential states are illustrated. **Left**: Osimertinib suppresses EGFR-driven PI3K–AKT–mTOR and RAS–RAF–MEK–ERK signaling, inducing apoptosis. **Center**: Epigenetic TROP2 silencing removes an inhibitory brake on IGF-1R, enabling EGFR-independent survival via the IGF-1–AKT–β-catenin–SLUG axis (primary resistance). **Right**: Under sustained osimertinib pressure, drug-tolerant persister cells adaptively re-express TROP2, amplifying IGF-2–IGF-1R–AKT signaling and activating NF-κB—providing the biological rationale for anti-TROP2 ADC therapy (Dato-DXd). Abbreviations: ADC, antibody-drug conjugate; AKT, protein kinase B; Dato-DXd, datopotamab deruxtecan; DTP, drug-tolerant persister; EGF, epidermal growth factor; EGFR, epidermal growth factor receptor; ERK, extracellular signal-regulated kinase; IGF, insulin-like growth factor; IGF-1R, insulin-like growth factor 1 receptor; MEK, mitogen-activated protein kinase kinase; mTOR, mechanistic target of rapamycin; NF-κB, nuclear factor kappa-B; PI3K, phosphoinositide 3-kinase; SLUG, SNAI2 transcription factor; TKI, tyrosine kinase inhibitor; TROP2, trophoblast cell-surface antigen 2.

One of the most frequent off-target resistance mechanisms to Osimertinib is the bypass activation of parallel receptor tyrosine kinases, by IGF-1R and IGF-2R, which maintain downstream PI3K/AKT and MAPK/ERK signaling despite EGFR blockade. Within this network, the IGF axis integrates both autocrine and paracrine stimuli from the tumor microenvironment (TME). Cancer-associated fibroblasts secrete IGF-1 and IGF-2, which bind to the respective receptors, sustaining AKT and ERK phosphorylation and promoting epithelial–mesenchymal transition (EMT) and cell survival (Figure 1) [7].

A pivotal role in this resistance mechanisms is played by TROP2, that is a 35 kDa transmembrane glycoprotein with extracellular EGF-like and thyroglobulin type-1 domains and a short intracellular tail containing a PIP_2_ binding and Ser303 phosphorylation motif. This structure allows TROP2 to act as a signal transducer, influencing Ca^2+^ flux, PKC and MAPK activation [11,12]. In normal epithelium, TROP2 contributes to cell adhesion and controlled proliferation in tumors, while in tumors its regulation is complex and context-dependent. Originally discovered in trophoblastic cells, TROP2 is overexpressed across many solid tumors and has been functionally implicated in multiple intracellular pathways. Although its oncogenic role remains less characterized than that of other pivotal signaling cascades, growing evidence has elucidated its crosstalk with more extensively studied oncogenic pathways. TROP2 is transcriptionally regulated by a complex network of transcription factors, with HNF4A serving as the major hub alongside TP63 and FOXM1, and its overexpression stimulates tumor growth via ERK/MAPK and cyclin D1 signaling, with downstream activation of NF-κB, STAT1, STAT3 and cyclin-dependent pathways [11]. TROP2 also functions as an intracellular calcium signal transducer and participates in EpCAM-mediated signaling [11].

Notably, negative impact of TROP2 on tumor growth has also been reported, particularly through suppression of IGF1-R signaling [11]. In lung adenocarcinoma, TROP2 may be silenced through promoter hypermethylation and loss of heterozygosity [13]. Restoration of TROP2 expression reduces AKT/ERK phosphorylation and inhibits proliferation, while knockdown enhances tumor growth [13]. Mechanistically, TROP2 binds IGF-1 and prevents its activation of IGF-1R, thereby blocking the IGF-1R/AKT/β-catenin/SLUG axis [5]. Consequently, loss of TROP2 removes a natural inhibitory brake on IGF-1R, enabling EGFR-independent survival signaling and potentially contributing to primary TKI resistance [13] (Figure 1).

On the other hand, TROP2 overexpression has also been associated with EGFR-mutated NSCLC resistance to gefitinib. In a cohort of 164 NSCLC patients, high TROP2 expression was detected in 82.1% of EGFR-mutant tumors compared with only 23.3% of EGFR wild-type cases, and EGFR-mutated patients with concomitant TROP2 overexpression was associated with worse overall survival (OS) [6]. Consistently, TROP2 protein levels were found to be higher in gefitinib-resistant PC-9/GR cell lines compared with parental sensitive cells, and its experimental overexpression directly increased resistance to gefitinib in vitro, while knockdown restored sensitivity [6].

It has been demonstrated that TROP2 interacts physically with IGF-2R, amplifying the IGF2–IGF-1R–AKT axis, enhancing proliferation, and remodeling TME through macrophage recruitment and cytokine secretion. Co-targeting TROP2 and IGF-1R with linsitinib reversed resistance in xenograft models, underscoring a cell-intrinsic and TME-mediated mechanism of acquired resistance [6]. Recent single-cell transcriptomic studies also revealed that EGFR inhibition is enriched for TROP2-high DTP cells, representing a minimal residual disease state with stem-like and embryonic signatures [14]. Baldacci et al. showed that TROP2 expression is markedly increased in DTPs following Osimertinib treatment both in patient samples and xenograft models. These observations suggest that TROP2 upregulation may serve as a marker of persistence rather than a driver mutation [14]. More recently, proteomic analyses among a subgroup of patients prospectively enrolled in the ELIOS study demonstrated high TROP2 expression in EGFR-mutated patients both at baseline and in tissue collected after progression on Osimertinib [15], while an increase in TROP2 protein expression on the cell surface was demonstrated both in vivo and in vitro after exposure to Osimertinib; in this study, TROP2 cell surface levels returned to baseline levels when Osimertinib was stopped [16].

Overall, as shown in Figure 1, TROP2 plays a dynamic role in EGFR-mutant NSCLC: it might be epigenetically silenced in early tumor evolution—thereby removing a natural brake on IGF-1R signaling and contributing to primary TKI resistance—and subsequently re-expressed and enriched on the surface of DTP cells under sustained Osimertinib pressure, rendering it a stable and accessible therapeutic target precisely in the resistant state [6,11,13]. Taken together, these findings suggest that during prolonged therapeutic pressure, re-expression or hyperactivation of TROP2 promotes IGF-2-IGF-1R crosstalk, activates NF-kB and cyclin D1, and remodels the tumor microenvironment, functioning as a molecular bridge between EGFR and IGF signaling that coordinates resistance pathways and stromal adaptation [6,11,14,15,16].

In early-phase clinical and translational studies of TROP2-directed ADCs, EGFR-mutant tumors appeared to derive preferential benefit, potentially reflecting enhanced ADC internalization and intracellular payload delivery associated with oncogenic EGFR signaling—a hypothesis currently under evaluation [17,18]. In this context, recent preclinical in vivo and in vitro data demonstrated specific activity of Dato-Dxd, a TROP 2-directed ADC in EGFR-mutated models, independently of TROP2 expression level [19]. A candidate explanation for this differential sensitivity resides in the NRF2 signaling pathway. The NRF2 transcriptional signature, which encompasses ABCC1, a known intracellular efflux transporter for the Dato-DXd payload, correlated with reduced sensitivity to both free DXd and Dato-DXd across NSCLC cell lines. EGFR-mutated models exhibited a trend towards lower NRF2/ABCC1 expression compared with EGFR wild-type counterparts, consistently with greater intracellular payload retention and cytotoxic efficacy in this molecular subtype [19]. Furthermore, Osimertinib pre-treatment of EGFR-mutated cell lines enhanced Dato-dxd internalization without significantly increasing surface TROP2 binding, a phenotype requiring sustained Osimertinib exposure to maintain elevated cell-surface TROP2 levels [19].

A further mechanism likely to amplify the therapeutic advantage of TROP2-directed ADCs in EGFR-mutated NSCLC is the bystander antitumor effect. Following TROP2-mediated internalization of the ADC and intracellular linker cleavage, the released payload diffuses across cell membranes into adjacent tumor cells regardless of their TROP2 expression, inducing DNA damage and cell death beyond the primary target population [20,21]. This mechanism has been formally demonstrated in preclinical lung cancer xenograft models using admixed TROP2-positive and TROP2-negative cell lines: Dato-DXd elicited potent tumor growth inhibition across all mixed-population models tested. While DXd localization was confined to TROP2-expressing cells, immunohistochemical evidence of γH2AX induction, a marker of double-strand DNA damage, was observed in TROP2-negative cells adjacent to TROP2-positive ones [22]. Critically, this bystander killing effect was preserved even when TROP2-positive cells represented only a minority of the tumor mass, indicating that partial antigen expression does not preclude full cytotoxic coverage of the tumor bulk [22,23]. This property is of particular relevance in the context of EGFR-mutated NSCLC, where TROP2 surface expression is characteristically heterogeneous, varying across subclones, evolving under therapeutic pressure, and further modified by epigenetic regulation. In addition, DTP cells may downregulate TROP2 while remaining spatially contiguous with TROP2-high residual clones [11,13,14].

In this setting, the bystander effect transforms TROP2 heterogeneity from a potential limitation into a therapeutically permissive condition, enabling payload delivery to antigen-low or antigen-negative cells via a paracrine cytotoxic mechanism.

## 3. Clinical Development of TROP2-Directed ADCs in EGFR-Mutated NSCLC

On the basis of TROP2 overexpression in lung cancer, TROP2-directed ADCs are under evaluation in the clinical context. In particular, three ADCs targeting TROP2 are being investigated in the context of EGFR-mutated NSCLC.

Dato-DXd is composed of a humanized anti-TROP2 IgG1 monoclonal antibody (datopotamab) conjugated via a tetrapeptide-based, enzymatically cleavable linker to DXd, an exatecan-derived topoisomerase I inhibitor, with an average drug-to-antibody ratio (DAR) of 4. The linker is designed to be stable in systemic circulation and selectively cleaved by lysosomal enzymes following ADC internalization, releasing a highly membrane-permeable payload that mediates potent bystander cytotoxicity in adjacent TROP2-negative cells [20]. Consistent with this mechanism, in the TROPION-PanTumour01 study, antitumor responses were observed regardless of TROP2 expression level by immunohistochemistry [18].

Sac-Gov is composed of the humanized anti-TROP2 monoclonal antibody hRS7 (IgG) conjugated to SN-38, the active metabolite of irinotecan and a topoisomerase I inhibitor, via a hydrolysable CL2A linker. Sac-gov compensates for the more moderate potency of SN-38 with a higher DAR of approximately 7.6 SN-38 molecules per IgG [24].

Sac-TMT is an ADC composed of a humanized anti-TROP2 IgG1 monoclonal antibody conjugated to KL610023, a belotecan-derived topoisomerase I inhibitor, via a sulfonylpyrimidine-CL2A-carbonate linker at a DAR of approximately 7.4. The linker employs an irreversible covalent binding via nucleophilic aromatic substitution, with high plasma stability, resulting in prolonged conjugate circulation time and enhanced intratumoral payload accumulation in preclinical models; however, prolonged systemic exposure also increases the potential for distribution to antigen-expressing normal tissues, a general mechanism of on-target off-tumor toxicity relevant to all TROP2-directed ADCs [25,26].

These three constructs thus span the mechanistic spectrum of linker design, from enzymatic cleavage (Dato-DXd) to pH-dependent hydrolysis (Sac-Gov) to irreversible covalent conjugation (Sac-TMT). This gradient has direct pharmacological consequences [27]. A first determinant is payload diffusibility: both DXd and SN-38 rank among the few ADC payloads whose lipophilicity and membrane permeability permit the bystander killing of adjacent antigen-low cells, a property central to the activity in heterogeneously TROP2-expressing tumors [28,29].  A second is plasma stability, which increases across the series and shapes systemic exposure: the short circulating half-life of Sac-Gov (~16 h) contrasts with the high stability and prolonged circulation conferred by the covalent Sac-TMT linker [28]. Critically, these properties translate into distinct toxicity phenotypes. As detailed in Section 3.2, the highly stable Sac-TMT linker, by limiting intracellular payload release in normal tissues, is associated with a myelosuppression-dominant profile that may in part reflect prolonged systemic exposure, whereas the internalization-dependent lysosomal cleavage of the Dato-DXd linker is mechanistically consistent with its epithelial (mucosal and ocular) toxicities. These structure–activity relationships indicate that, even within a shared target and payload class, linker chemistry is a key modulator of the therapeutic index and a rational lever for ADC optimization [28].

Here, we summarize clinical data about TROP2-directed ADCs in EGFR-mutated NSCLC.

Table 1 summarizes results available from clinical trials, with specific focus on included EGFR-mutated patients, while Table 2 includes ongoing clinical trials in EGFR-mutated NSCLC.

### 3.1. Available Clinical Data in EGFR-Mutated NSCLC

While many clinical trials are still ongoing, early-phase clinical trials data as well as results from three randomized phase III clinical trials are already available to shed light on future direction in the development of TROP-2-directed ADC in EGFR-mutated NSCLC.

After early-phase studies (Tropion-PanTumor01 and Tropion-PanTumor 02) showed response rate (ORR) ranging from 22% to 26% across doses and disease control rates consistently above 70% in NSCLC patients, Dato-DXd is currently being investigated in several different clinical settings [18]. The drug has therefore been primarily investigated in advanced patients progressing to platinum-based treatment and, in the presence of actionable genomic alterations (AGAs), also to targeted agents. TROPION-Lung05 is a phase II study specifically focusing on patients with AGAs in which Dato-DXd was administered at the dosage of 6 mg/kg every three weeks and achieved an ORR of 35.8% (95% CI: 27.8–44.4) in the overall population (137 patients) and 43.6% (95% CI: 32.4–55.3) among 78 patients with EGFR mutations. The patients included were heavily pre-treated, and the vast majority of the overall study population had previously received more than three lines of systemic treatment [30].

On the other hand, the only phase III randomized trial testing Dato-DXd whose results are currently available is characterized by high heterogeneity in the study population since it included both squamous and non-squamous histology as well as both oncogene-addicted and non-oncogene-addicted disease. The phase III TROPION-Lung01 randomized patients progressing on platinum-based chemotherapy (after one or two lines of targeted agents in the presence of AGAs) to receive standard treatment with docetaxel or Dato-DXd 6 mg/kg every three weeks. Co-primary endpoints were progression-free survival (PFS) and OS. The trial met the PFS endpoint, while the OS benefit was not statistically significant in the molecularly unselected study population. The trial demonstrated higher ORR with Dato-DXd compared with docetaxel (26.4% vs. 12.8%), improved disease control rate (DCR) (77.3% vs. 64.9%), and longer median PFS in both the overall study population (4.4 vs. 3.7 months, HR: 0.75, 95% CI: 0.62–0.91, *p*:0.004) and in the subgroup of patients carrying AGAs (5.7 vs. 2.6 months, HR: 0.63, 95%, CI 0.51–0.79). Although the absolute PFS gain in the overall population was modest, the benefit appeared more relevant in non-squamous histology and in patients with AGAs.

Since the majority of AGAs were EGFR mutations, a pooled analysis including 117 EGFR-mutant patients enrolled in Tropion-lung 01 (*N*: 39) and in Tropion-lung 05 (*N*: 78) and treated with Dato-DXd was performed. Although the population was heavily pre-treated (a median of three lines of previous), ORR was 43% (95%CI: 34–52%) and DCR 86% (95% CI: 79–92%). The median PFS was 5.9 (95%CI: 5.4–8.2) months and median OS 15.6 (95% CI: 13.1–19) months. Following these data, the FDA granted accelerated approval to the drug for EGFR-mutated NSCLC previously receiving previous TKI and platinum-based chemotherapy [17]. In parallel, recent results from the phase 2 ORCHARD study seem to be consistent with a potential increasing role of the drug in EGFR-mutated patients progressing on Osimertinib. The trial was designed to assess novel post-progression combination therapies in patients who progressed on first-line Osimertinib. Different arms of treatment were designed according to the specific mechanism of acquired resistance detected at the time of progression in tissue rebiopsy. Patients without new druggable co-alterations at the time of progression and without histological transformation were treated with Dato-DXd and Osimertinib. Two different dosages were included (4 and 6 mg/kg every three weeks): in the two cohorts, median PFS was 9.5 (95% CI: 7.2–9.8) months and 11.7 (95% CI: 8.3–21.7) months, respectively, while median duration of response was 6.3 (95% CI 3.8–8.1) and 20.5 (95% CI 6.2-not calculable [NC]). ORR was similar between the two cohorts (36 vs. 43%) even if faster time to response and greater target lesion shrinkage were found in the 6 mg/kg arm [31].

Clinical randomized trials focusing on the role of Dato-DXd in EGFR-mutated patients are ongoing. Tropion-lung 14 is a clinical trial designed to compare Osimertinib versus Osimertinib plus Dato-DXd in a first-line setting, while Tropion-lung 15 enrolls patients progressing to first-line Osimertinib and randomizes them to receive either standard platinum-based chemotherapy or Dato-DXd or Dato-DXd plus Osimertinib and will let us draw conclusions about the superiority of dato-dxd versus platinum-based chemotherapy in patients progressing to Osimertinib and shed light on the potential contribution of continuing Osimertinib with Dato-DXd in patients progressing to Osimertinib [32,33].

Sac-TMT is another TROP-2 ADC that demonstrated consistent activity and efficacy signals from phase I/II through phase III development. The results of phase I (KL264-01) and II (SKB264-II-08) were published together in 2024, and in both single-arm trials, ORR and median PFS in the overall pre-treated population were considered promising, but greatest potentialities were observed in the EGFR-mutated population [34]. In a phase I study, 22 pre-treated patients had EGFR sensitizing mutations and achieved an ORR of 55% (95% CI: 32–76) and median PFS of 11.1 (95% CI: 5.7–12.9) months. In a phase II study, 64 EGFR-mutated patients were included; 32 of them already received both EGFR-TKI and platinum-based chemotherapy [34]. Patients were treated with Sac-TMT 5 mg/kg every two weeks and EGFR-mutated patients achieved ORR of 34% (95% CI: 23–47) with DCR of 84% (95% CI: 73–92) and median PFS of 9.3 (95% CI: 7.6–11.4) months [34]. The phase III trial OptiTROP-Lung03 compared Sac-TMT with docetaxel in previously treated EGFR-mutated NSCLC and confirmed its superiority over standard chemotherapy, with a significantly higher ORR (45.1% vs. 15.6%) and a clinically meaningful improvement in median PFS (6.9 vs. 2.8 months). The included patients had already received both EGFR TKI and platinum-based chemotherapy [35]. More importantly, the phase III trial OptiTROP-Lung04 compared treatment with Sac-TMT 5 mg/kg every two weeks with platinum-based chemotherapy in EGFR-mutated NSCLC progressing to EGFR-TKI. The primary endpoint was PFS by independent review and was met with high statistical and clinical significance. Median PFS was 8.3 months (95% CI: 6.7–9.9) for patients treated with the experimental arm versus 4.3 months (95% CI: 4.2–5.5), HR: 0.49 (95% CI: 0.39–0.62). PFS-rate at 12 months was 32.3% (95% CI: 25.5–39.2) versus 7.9% (95% CI, 4.4 to 12.8). OS, as a secondary endpoint, was also met. Median OS was not reached in the Sac-TMT arm versus 17.4 months, with HR of 0.60 (95% CI, 0.44 to 0.82). OS at 18 months was 65.8% (95% CI, 58.3 to 72.3) with Sac-TMT and 48.0% (95% CI, 40.2 to 55.4) with chemotherapy [36]. Although both OptiTROP-Lung03 and OptiTROP-Lung04 included only Chinese centers, the results clearly indicate a potential role for anti-TROP2 ADC in the therapeutic pathways of EGFR-mutated NSCLC.

Consistently, a recent meta-analysis of 11 phase III randomized trials (*n* = 3650) evaluated second-line different treatment strategies in patients with EGFR-mutant NSCLC following TKI failure. Among the evaluated regimens, SacTMT demonstrated significant improvements in both PFS (HR 0.49) and OS (HR 0.60) compared with chemotherapy and chemo-immunotherapy (HR 0.64 for PFS; HR 0.68 for OS), without increasing severe toxicity probability [37]. Overall, these findings position Trop-2-directed ADCs, and specifically SacTMT, as a promising therapeutic strategy in the post-TKI setting for EGFR-mutant NSCLC, with a potentially favorable balance between efficacy and tolerability [37].

Several clinical trials are currently opening, although they have not yet begun recruiting, with the aim of defining the role of sacTMT-containing combination therapies in EGFR-mutant non-small cell lung cancer, as well as evaluating their potential application in early-stage disease [38,39,40].

Fewer data are available for Sac-gov in EGFR-mutated NSCLC settings. EVOKE 01 is a randomized phase III trial that compared the Sac-gov with docetaxel in previously treated advanced NSCLC. Notably, the population in this study was not molecularly selected even though a previous targeted treatment for AGAs was a stratification factor. The primary endpoint was OS and was not met in the overall study population, although it was numerically superior in the experimental arm. Median PFS was 4.1 versus 3.9 months with HR of 0.92 (95% CI: 0.77 to 1.11). Only 19 patients previously treated with EGFR-TKI were included [41].

### 3.2. Safety and Tolerability

Although common features are present, specific anti-TROP2 ADCs exhibit distinct toxicity profiles which are strongly influenced by the type of payload, linker stability, drug dosage, and patient characteristics [42,43]. Toxicity can be broadly classified as on-target, when related to antigen expression in normal tissues, and off-target, when arising from non-specific uptake or systemic payload release in non-target-cells [26].

The clinical toxicity profile of ADCs primarily relates to the payload component, with dose-limiting toxicities often shared by different ADCs delivering the same cytotoxic payload regardless of the antigen targeted. Ocular toxicity can arise through two mechanistically distinct pathways. The off-target mechanism is most characteristic of MMAF- and DM4-containing constructs, in which non-specific cellular uptake leads to intracellular accumulation of charged catabolites in the corneal epithelium. On the other hand, the on-target mechanism is directly relevant to TROP2-directed ADCs, in which ADC binding to TROP2 expressed on corneal epithelial cells drives receptor-mediated internalization and local intracellular payload release [26]. The latter mechanism is consistent with the clinically observed ocular toxicity of Dato-DXd, whose tetrapeptide linker undergoes lysosomal cleavage following TROP2-mediated internalization, and with the virtual absence of ocular events with Sac-TMT, whose highly stable sulfonylpyrimidine linker substantially limits intracellular payload release in normal tissues [26].

Here, we summarize the toxicity profiles of ADC emerging from trials involving patients with EGFR-mutated NSCLC.

**Figure 2:**
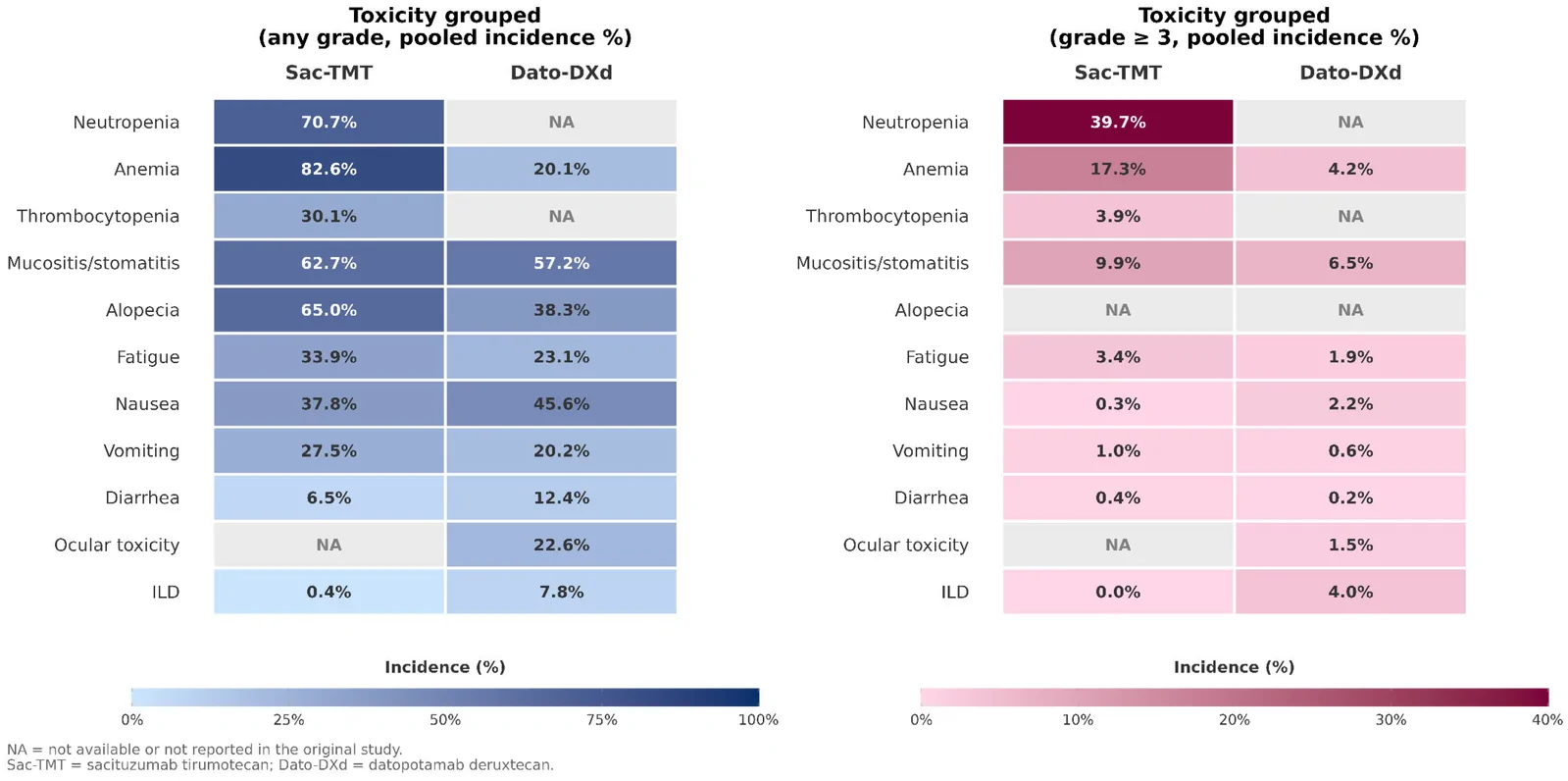
Pooled toxicity heatmap of sacituzumab tirumotecan (Sac-TMT) and datopotamab deruxtecan (Dato-DXd) in EGFR-mutated NSCLC. Pooled incidence of treatment-related adverse events for any grade (left panel) and grade ≥ 3 (right panel). Color intensity reflects incidence rate. Sac-TMT displays a myelosuppression-dominant profile (neutropenia 70.7%, anemia 82.6%), whereas Dato-DXd is characterized by epithelial toxicity (mucositis 57.2%, ocular toxicity 22.6%) and a clinically relevant risk of ILD/pneumonitis (any grade 7.8%, grade ≥ 3 4.0%). Mucositis/stomatitis represents a shared class effect across both agents. Abbreviations: Dato-DXd, datopotamab deruxtecan; ILD, interstitial lung disease; NA, not available or not reported in the original study; NSCLC, non-small cell lung cancer; Sac-TMT, sacituzumab tirumotecan.

Figure 2 reports pooled crude incidences derived from our own trial-level extraction and represents a descriptive summary. Safety data were pooled from the NSCLC trials and dose cohorts of each agent (Sac-TMT: KL264-01, SKB264-II-08, OptiTROP-Lung03, OptiTROP-Lung04, and a phase 2 cohort with uncommon EGFR mutations; Dato-DXd: TROPION-PanTumor01 [4/6/8 mg/kg], TROPION-PanTumor02, TROPION-Lung05, TROPION-Lung01, ORCHARD). For each adverse event, incidence was pooled at the patient level as the number of event-positive patients divided by the summed population of the reporting studies only, so estimates are implicitly weighted by sample size. Studies not reporting a given event were excluded from its denominator rather than counted as zero; consequently, denominators vary across events, and pooled rates for infrequently reported events may be overestimated, so cross-agent comparison of individual toxicities should be interpreted with caution.

In particular, across the pooled datasets, treatment-related adverse events (TRAEs) of any grade were reported in 98.9% of patients treated with Sac-TMT and 92.5% of those receiving Dato-DXd, while grade 3 toxicities occurred in 61.7% and 35.4% of patients, respectively (Figure 2). For Sac-TMT, the pooled data clearly identifies hematologic toxicity as the dominant adverse-event class, as summarized in Figure 2. Neutrophil count decrease was observed in 70.7% of patients overall, with 39.7% experiencing grade 3 events, while anemia affected 82.6% including 17.3% grade 3 cases. These findings are consistent with safety signals reported in the KL264-01 study, the SKB264-II-08 expansion cohort, and the randomized phase III OptiTROP-Lung03 and support the notion that myelosuppression represents the principal toxicity driver of Sac-TMT. Importantly, despite the high incidence of neutropenia reported in Figure 2, febrile neutropenia and permanent discontinuation of treatment due to hematologic events remained uncommon across studies [34,36]. Among non-hematologic adverse events, oral mucosal toxicity emerges as a relevant shared adverse event, with stomatitis/mucositis reported in 62.7% of patients treated with Sac-TMT, although grade ≥ 3 events were limited to 9.9%, confirming a predominantly low-to-moderate severity profile. This toxicity did not require definitive dose interruptions and a reduction in 14% [17]. Notably, pulmonary toxicity was virtually absent across all Sac-TMT trials, with a pooled interstitial lung disease (ILD)/pneumonitis incidence of only 0.4% and no grade ≥ 3 events.

In contrast, the pooled toxicity profile of Dato-DXd shows a qualitatively distinct pattern, dominated by epithelial rather than hematologic adverse events, as detailed in Figure 2. Oral mucositis represents the most frequent toxicity, occurring in 57.2% patients, with 6.5% experiencing grade 3 events, underscoring mucositis as a class-defining and shared toxicity for both TROP2-directed ADCs. Hematologic toxicity was comparatively infrequent with anemia observed in 20.1% of patients overall, with only 4.2% grade 3 events, corroborating trial-level data indicating a lower burden of severe myelosuppression relative to Sac-TMT.

A distinctive safety feature of Dato-DXd is the occurrence of ocular toxicity, which in the pooled dataset comprised a spectrum of predominantly ocular surface adverse events, including lacrimation disorders, dry eye syndrome, conjunctivitis, and keratitis [18,21,30] (Figure 2). These events were largely low-grade in severity, indicating a manageable but agent-specific ocular safety signal. In addition, unlike SacTMT, Dato-DXd is associated with a reproducible risk of interstitial lung disease (ILD)/pneumonitis. In our pooled dataset, ILD occurred in a clinically relevant proportion of patients (7.8%), with grade ≥ 3 events reported in 4.0% of cases, including rare fatal outcomes (Figure 2).

The divergent toxicity phenotypes of Sac-TMT and Dato-DXd are also reflected in their respective patterns of dose modification and treatment discontinuation.

In our pooled dataset, dose interruptions and reductions were substantially more frequent with Sac-TMT than with Dato-DXd (40.4% vs. 20.3% and 30.8% vs. 20.8%, respectively), consistent with the need for active management of hematological toxicity to sustain treatment continuity. Notably, however, permanent discontinuation due to adverse events was not reported in any patient in the pooled Sac-TMT dataset. In contrast, treatment discontinuation occurred in 10.5% of Dato-DXd-treated patients in the pooled analysis, a figure consistent with individual trial data from TROPION-Lung01 (8.1%) and TROPION-Lung05 (5.1%), where ILD/pneumonitis emerged as the leading cause of permanent withdrawal [21,30].

In the EVOKE-01 trial, the safety profile of Sac-Gov in the overall previously treated NSCLC population was consistent with the known class effects of topoisomerase I inhibitors. Hematological toxicity and diarrhea represented the predominant adverse events, while ILD and ocular toxicity were not reported as clinically relevant [41]. This distinct toxicity phenotype reflects the structural features of Sac-Gov and differs from both Dato-DXd and Sac-TMT, reinforcing the view that toxicity in TROP2-directed ADCs is predominantly payload-driven rather than target-driven [26].

Taken together, the tabulated pooled data highlight clearly divergent toxicity phenotypes among TROP2-directed ADCs. Sac-TMT and Sac-gov are characterized by a myelosuppression-dominant toxicity profile, whereas Dato-DXd displays a predominantly epithelial toxicity pattern, marked by ocular surface events and a clinically relevant risk of ILD. Notably, oral mucositis emerges as a frequent and shared adverse event across agents, occurring in a substantial proportion of treated patients, although it is predominantly low-grade and rarely dose-limiting, underscoring the need for proactive supportive care strategies irrespective of the selected TROP2-directed ADC [44].

The management of ADC toxicities represents a new challenge that requires training of clinicians as well as patients’ and caregivers’ education, especially for the management of chronic low-grade toxicities (stomatitis, ocular toxicity, hematological dyscrasias) that can compromise quality of life and lead to unnecessary dose reductions or interruptions. During treatment, regular hematological and clinical monitors are required and stomatitis protocols are recommended (oral hygiene, topical therapies, analgesics). A prospective study in lung and breast cancer has been planned, focusing on stomatitis and the use of prophylactic dexamethasone mouthwash within the first 12 weeks of study treatment (NCT07357597) [45].

## 4. Focus on ILD

As previously discussed, ILD represents an emerging toxicity factor and the most important cause of permanent discontinuation, while drug exposure is associated with efficacy. In this context, understanding potential risk factors and optimizing early detection represents a key factor in TROP2-directed ADC clinical development. From a pathogenetic standpoint, we first consider data collecting with other ADCs. In particular, animal studies concerning ILD induced by trastuzumab deruxtecan, an ADC directed against HER2, showed that damage occurs in HER2-negative alveoli, while HER2-positive areas are unaffected. This indicates that toxicity might be independent of the target. The main cause appears to be a nonspecific uptake of ADC by alveolar macrophages, which express cathepsin B, an enzyme capable of cleaving the linker and releasing the DXd payload, causing direct cytotoxicity. However, circulating free DXd alone does not explain the observed toxicity, while immune cell-mediated off-target effects are supposed to play fundamental pathogenic role [46].

Currently, no clear data are available on biomarkers able to predict the risk of TROP2-directed ADC-induced pulmonary toxicity in lung cancer. As a first step, potential synergistic factors in the development of toxicity should be considered.

From a clinical standpoint, baseline clinical and respiratory functional assessment, including careful evaluation of thoracic CT-scan, can help identify pre-existing chronic pulmonary diseases. In addition, previous or concomitant anti-cancer treatment carrying an intrinsic risk or pulmonary toxicity must always be factored in when assessing individual patient risk and when interpreting toxicity data from clinical trials. In particular, previous thorax radiotherapy and previous or concomitant treatment with Osimertinib are likely to play a synergistic role in increasing pulmonary toxicity risk.

Osimertinib-induced ILD has an incidence of approximately 4%; therefore, when interpreting and comparing the pulmonary toxicity profiles across different ADC-based regimens, the proportion of patients who previously or concomitantly received Osimertinib must always be factored in, as it may substantially contribute to the observed ILD burden beyond what is attributable to the ADC itself [47].

In the ORCHARD study, patients treated with the combination of Osimertinib and Dato-DXd experienced adjudicated ILD/pneumonitis in 3% of cases in the 4 mg/kg cohort and in 15% of cases in the 6 mg/kg cohort, with no ILD-related fatal events reported in either arm; notably, two thirds of ILD cases occurred in Asian patients despite this subgroup representing only one-third of the overall population [31].

A comparable amplification of pulmonary toxicity risk is observed when Dato-DXd is combined with immune-checkpoint inhibitors. In the phase Ib TROPION-Lung02 study evaluating Dato-DXd plus pembrolizumab in non-AGA NSCLC, adjudicated ILD or pneumonitis occurred in 17.1% and 25% of patients receiving doublet and triplet therapy respectively, though severe events remained infrequent and no fatal ILD was reported in either arm [48].

In addition to risk identification, early recognition plays an essential role in proper management, with the aim of avoiding severe toxicity and permanent discontinuation, if necessary.

Assessment by an experienced multidisciplinary team, including review of thoracic CT scans, is essential to identify pre-existing lung parenchyma alteration—even when asymptomatic at baseline—and to detect early changes during ADC treatment.

The issue probably deserves specifically addressed protocols involving remote expert meetings, and artificial intelligence-based methods might be developed.

## 5. Biomarkers: What We Know up to Now

As previously stated, the integration of TROP2-targeting ADCs is yielding promising results with the potential to reshape treatment paradigms. However, the optimal strategy for patient selection remains unclear, as there is currently no validated biomarker to reliably predict which patients will derive the greatest benefit from therapy. This critical step underscores the urgent need to define predictive biomarkers. Consequently, the landscape of investigated biomarkers is rapidly evolving, shifting from an initial focus on assessing TROP2 expression through various techniques to the inclusion of additional predictive markers.

Historically, the development of TROP2-directed ADCs was guided by the premise that target overexpression would predict response. However, this hypothesis proved inadequate. Across different trials, the efficacy of TROP2-ADCs was independent of TROP2 protein or mRNA levels when ADCs were tested in patients unselected either for histology or for molecular features [49,50,51].

A pivotal breakthrough came from exploratory analyses which identified that patients with EGFR mutations derived a significant greater benefit. This was clinically observed in a pooled analysis of 117 EGFR-mutated patients from the TROPION-Lung01 and TROPION-Lung05 trials [17]. This evidence was then corroborated by similar findings from other phase trials with Sac-TMT [36]. Within the EGFR-mutant population, the level of TROP2 expression, quantified via immunohistochemistry-determined H-score, is re-emerging as a potential biomarker for response durability. Prespecified subgroup analysis from KL264-01 and SKB264-II-08 trials revealed that patients with high TROP 2 expression (defined as a H-score >200), compared to those with a lower TROP 2 expression, had similar ORR with Sac-TMT, but PFS was significantly longer in the high-expression group (10.9 vs. 7.2 months, *p* = 0.020) [34]. This suggests that while a minimal threshold of TROP2 is sufficient for initial clinical activity, higher target density may facilitate more sustained target engagement, leading to prolonged disease control.

Given that ADCs require cellular internalization to release their cytotoxic payload, the subcellular distribution of its target, TROP2, may critically influence therapeutic efficacy. In this context, an exploratory analysis of the phase III TROPION-Lung01 trial investigated the predictive value of TROP2 as assessed by a proprietary computational pathology platform known as quantitative continuous scoring (QCS). This fully supervised platform analyses digitized tissue samples to precisely quantify the subcellular localization of TROP2, generating a normalized membrane ratio (NMR) for each tumor cell. Tumors were classified as TROP2-QCS biomarker positive if the majority (≥75%) of cells exhibited a low membrane-to-cytoplasm ratio (≤0.56), indicating predominant cytoplasmic localization. In this predefined subgroup, which comprised 60% of the biomarker-evaluable population, Dato-DXd demonstrated a markedly enhanced benefit over docetaxel, reducing the risk of disease progression or death by 43% (median PFS 6.9 vs. 4.1 months; HR 0.57 [95% CI 0.41–0.79]), a much greater magnitude of effect than observed in the overall trial population [52,53]. Notably, the magnitude of benefit observed in the TROP2-QCS-high subgroup appears broadly comparable to that reported in patients with AGA-positive NSCLC [21].

The mechanistic basis of QCS as a predictive platform has been further elucidated in translational studies demonstrating that Dato-DXd requires binding to membrane-localized TROP2 for internalization, while cytoplasmatic TROP2 is not directly accessible for ADC engagement [54]. Cells exhibiting predominantly cytoplasmic TROP2 distribution, the NMR-positive phenotype, appear to reflect a state of active receptor cycling in which TROP2 is continuously internalized from the membrane and accumulates in the cytoplasmic compartment. QCS-NMR therefore captures TROP2 internalization dynamics rather than static expression level, providing a more biologically informed basis for patient selection than conventional histochemical-score methodology [54]. Consistently, an independent retrospective exploratory analysis of 76 treatment-naïve patients from the phase IB TROPION-Lung02 study, in which Dato-DXd was combined with pembrolizumab with or without platinum-based chemotherapy, similarly demonstrated a numerical trend toward improved PFS and OS in NMR-positive compared with NMR-negative patients (median PFS 12.0 versus 8.1 months; HR 0.62, 95% CI 0.35–1.10) [48]. These data provide cross-trial support for the QCS predictive platform across different treatment combinations and clinical settings in non-AGA NSCLC [48].

Of note, QCS cut-points were developed and validated exclusively in non-squamous/non-AGA NSCLC. When applied to 68 evaluable EGFR-mutated patients from TROPION-Lung01 and TROPION-Lung05, TROP2 NMR status failed to show association with clinical outcome. Median PFS was virtually identical between NMR-positive and NMR-negative patients (HR 0.95, 95% CI 0.53–1.72; *p* = 0.88) [55]. A consistent absence of predictive signal was observed with Dato-DXd plus Osimertinib in the ORCHARD study, though interpretation is substantially limited by the very small number of NMR-negative patients available (*n* = 5) [55]. Taken together, these findings suggest that EGFR mutation status itself might represent an enrichment marker for Dato-DXd benefit, rendering QCS-based further stratification potentially redundant. Prospective evaluation in adequately powered, biomarker-annotated cohorts remains necessary before definitive conclusions can be drawn.

No evidence is available concerning the applicability of QCS-NMR to other TROP2-directed ADCs, and specific validation must be performed given the potential impact of different linker stability on the predictive role of NMR.

Other exploratory biomarkers that may be useful for further stratification are related to the payload specific pathways: high tumor expression of TOP1, the target of the SN-38 payload in SG, has been associated with improved outcomes in other malignancies [56]. Similarly, deficiencies in DNA damage response pathways have also been linked to a numerically greater benefit from SG in breast cancer, suggesting a possible synergy relevant to ADC payload mechanisms [57]. However, the clinical utility of these biomarkers in EGFR-mutant NSCLC remains unclear and requires prospective validation.

Future strategies will likely integrate this genomic selection with refined protein expression analysis and other molecular features to optimize the use of this potent therapeutic class.

## 6. Conclusions and Future Directions

Overall, the summarized biological and clinical data suggest a future role for TROP2-targeted ADCs in the treatment of EGFR-mutated NSCLC.

From a biological point of view, the framework for the use of TROP2-directed ADCs to eradicate residual and drug-tolerant clones in EGFR-mutated NSCLC includes target enrichment under selective pressure, preserved cell surface accessibility, and bystander-mediated coverage of heterogeneous tumor populations [21,30].

On the other hand, current clinical data already support a role for these agents in patients progressing on first-line Osimertinib, as confirmed by a recent meta-analysis [47]. even though randomized data in non-Asian populations are still awaited. In this context, the ORCHARD study as well as recent evidence from the COMPEL clinical trial support, from a clinical standpoint, the biological rationale for continuing EGFR inhibition beyond progression on first-line TKI [31,58]. The association of Osimertinib beyond progression and anti-TROP2 ADC is also corroborated by translational findings showing that TROP2 cell surface levels are affected by Osimertinib administration, therefore suggesting synergism in efficacy [16].

In this evolving context, defining the optimal positioning of TROP2-directed ADCs across lines of therapy becomes a priority.

The therapeutic scenario is getting crowded, and TROP-directed ADCs are likely to become one potential standard in second line setting, together with other options including both strategies driven by the specific resistance mechanism and strategies designed for unselected patients.

These competing options include distinct drug classes. Among non-ADC antibody strategies, the EGFR–MET bispecific amivantamab plus chemotherapy was the first regimen to improve PFS over chemotherapy after osimertinib progression in the phase III MARIPOSA-2 trial (median PFS 6.3 vs. 4.2 months; HR 0.48), albeit with substantial EGFR/MET-related toxicity [59]. The PD-1 × VEGF bispecific ivonescimab plus chemotherapy improved PFS over chemotherapy in EGFR-mutant patients progressing after a third-generation TKI in the phase III HARMONi-A trial (median PFS 7.06 vs. 4.80 months; HR 0.46) [60]. ADCs directed at targets other than TROP2 are also under investigation. Patritumab deruxtecan (HER3-DXd), which shares the deruxtecan payload and a tetrapeptide cleavable linker with Dato-DXd, produced durable responses in the phase II HERTHENA-Lung01 study (confirmed ORR 29.8%), but failed to improve OS versus chemotherapy in the phase III HERTHENA-Lung02 trial (median OS 16.0 vs. 15.9 months; HR 0.98), leading to withdrawal of its biologics license application [61,62]. The EGFR × HER3 bispecific ADC izalontamab brengitecan (Iza-bren) showed activity in an exploratory pooled analysis of phase I/II trials in EGFR-mutant NSCLC after TKI progression (confirmed ORR 47.4%, median PFS 6.9 months), although these data derive from uncontrolled, single-region cohorts and require randomized confirmation [63].

For this reason, correct sequencing of strategies even after first-line treatment is going to be a major challenge. Only specifically focused translational work might provide further information for personalizing treatment sequencing.

On the other hand, while ongoing studies are investigating the role of TROP2-directed ADC and Osimertinib also in first-line settings, their results will be analyzed in the new context of first-line EGFR-mutated diseases characterized by two new combination strategy options, which already demonstrated superiority over Osimertinib as a single agent, although characterized by higher toxicity [9,64].

A key challenge in NSCLC treatment in the coming years will therefore be the customization of therapy in EGFR-mutated NSCLC, with the dual aim of avoiding unnecessary toxicity in patients likely to derive prolonged clinical benefit from Osimertinib monotherapy, while intensifying treatment in those at risk of a relatively short PFS (and OS) with Osimertinib alone. To date, available information is limited to prognostic clinical and molecular markers, such as the presence of brain metastases and TP53 co-mutation, both established negative prognostic factors, though not directly predictive for treatment selection [9,64].

A further consolidated negative prognostic marker is the detection of EGFR-mutated circulating tumor DNA (ctDNA) in plasma at baseline, and even more so its persistence after the initiation of Osimertinib. [8,65,66,67] Liquid biopsy has also been proposed as a dynamic prognostic tool in other disease contexts, while results of interventional trials confirming potential roles in customizing treatment are still awaited [68,69]. An investigator-driven clinical trial specifically designed to address this question has been initiated in China, in which only patients with detectable ctDNA after three weeks of treatment will proceed to the combination of Sac-TMT and Osimertinib [70].

This represents an innovative approach to treatment selection, likely to be adopted and refined in future clinical trials. In parallel, a modern perspective on therapeutic strategies must also encompass clinical trials specifically designed to improve the management of treatment-related toxicity, as well as the implementation of multidisciplinary boards for the most challenging adverse events, such as stomatitis and ILD.

## Figures and Tables

**Table 1:** Clinical efficacy data of TROP2-directed antibody-drug conjugates in EGFR-mutated and unselected NSCLC populations across available trials. Results are reported for the overall study population (All) and for the EGFR-mutated subgroup (EGFR+) where available.

Treatment	Phase	*N* pts		ORR (%)		DCR (%)		PFS (mo)		OS (mo)		DOR (mo)		mFUP (mo)	Trial
		All	EGFR+	All	EGFR+	All	EGFR+	All	EGFR+	All	EGFR+	All	EGFR+		
Sacituzumab Tirumotecan (Sac-TMT)															
Sac-TMT 5 mg/kg q14d	I/II	43	22	—	55%	—	86%	—	11.1	—	21.8	—	—	26	NCT04152599 *(KL264-01)*
Sac-TMT 5 mg/kg q14d	II	64	64	—	34%	—	84%	—	9.3	—	NR	—	9.6	12.7	NCT05631262 *(SKB264-II-08)*
Sac-TMT 5 mg/kg q2w vs. Docetaxel 75 mg/m^2^ q3w	III	137	137	—	45.1 vs. 15.6%	—	—	—	6.9 vs. 2.8	—	NR	—	—	12.2	NCT05631262 *(OptiTROP-Lung03)*
Sac-TMT 5 mg/kg q2w vs. Pemetrexed + platinum	III	376	376	—	60.6 vs. 43.1%	—	—	—	8.3 vs. 4.3	—	NR vs. 17.4	—	8.3 vs. 4.2	18.9	NCT05870319 *(OptiTROP-Lung04)*
Datopotamab Deruxtecan (Dato-DXd)															
Dato-DXd 4 mg/kg q3w	**I**	50	50	—	22%	—	76%	—	4.3	—	12.9	—	—	—	NCT03401385 *(TROPION Pan-Tumor01)*
Dato-DXd 6 mg/kg q3w		50	50	—	26%	—	70%	—	6.9	—	11.4	—	—	—	
Dato-DXd 8 mg/kg q3w		80	80	—	23.8%	—	78.8%	—	5.2	—	10.5	—	—	—	
Dato-DXd 6 mg/kg q3w	I/II	40	4	45%	75%	85%	—	7.4	—	NA	—	8.3	—	8.1	NCT05460273 *(TROPION Pan-Tumor02)*
Dato-DXd 6 mg/kg q3w	II	137	78	35.8%	43.6%	78.8%	82.1%	5.4	5.8	13.6	—	7.0	7.0	15.2	NCT04484142 *(TROPION-Lung05)*
Dato-DXd 6 mg/kg q3w vs. Docetaxel 75 mg/m^2^ q3w	III	605	AGA+ ^†^	26.4 vs. 12.8%	37.5 vs. 8%	77.3 vs. 64.9%	93.8 vs. 44%	4.4 vs. 3.7	5.7 vs. 2.6	12.9 vs. 11.8	15.6 vs. 9.8	7.1 vs. 5.6	6.9 vs. NE	PFS: 10.9/9.6 OS: 23.1/23.1	NCT04656652 *(TROPION-Lung01)*
Dato-DXd 4 mg/kg + Osimertinib vs. Dato-DXd 6 mg/kg + Osimertinib	II	68	68	—	43 vs. 36%	—	—	—	9.5 vs. 11.7	—	immature	—	6.3 vs. 20.5	13.4/13.8	NCT03944772 *(ORCHARD)*
Sacituzumab Govitecan (Sac-Gov)															
SG 10 mg/kg d1,8 q21d vs. Docetaxel 75 mg/m^2^ q3w	III	299	6 ^‡^	13.7%	—	67.6%	—	4.1	—	11.1	—	6.7	—	12.7	NCT05089734 *(EVOKE-01)*

^†^ In TROPION-Lung01, the EGFR+ subgroup column refers to patients with non-squamous histology and actionable genomic alterations (AGA+), the majority of whom harbored EGFR mutations. ^‡^ In EVOKE-01, only six patients (2.0%) in the SG arm had documented EGFR alterations; no EGFR-mutated subgroup efficacy data were reported. **Abbreviations**: AGA, actionable genomic alteration; Dato-DXd, datopotamab deruxtecan; DCR, disease control rate; DOR, duration of response; EGFR, epidermal growth factor receptor; mFUP, median follow-up; mo, months; NCT, national clinical trial; NE, not estimable; NA, not available; NR, not reached; NSCLC, non-small cell lung cancer; ORR, objective response rate; OS, overall survival; PFS, progression-free survival; q2w, every 2 weeks; q3w, every 3 weeks; Sac-Gov, sacituzumab govitecan; Sac-TMT, sacituzumab tirumotecan; SG, sacituzumab govitecan.

**Table 2:** Ongoing phase III clinical trials investigating TROP2-directed antibody-drug conjugates (ADCs) in EGFR-mutated non-small cell lung cancer.

Trial	NCT Number	Investigational Product	Treatment Arms	Phase	Setting
Datopotamab Deruxtecan (Dato-DXd)					
TROPION-Lung14	NCT06350097	*Datopotamab Deruxtecan*	Arm A: Osimertinib 80 mg/day vs. **Arm B:** Osimertinib 80 mg/day + Dato-DXd 6 mg/kg q3w	III	1st line
TROPION-Lung15	NCT06417814	*Datopotamab Deruxtecan*	Arm A: Platinum-based doublet CT vs. Arm B: Dato-DXd 6 mg/kg q3w vs. Arm C: Dato-DXd 6 mg/kg q3w + Osimertinib 80 mg/day	III	Post-osimertinib
Sacituzumab Tirumotecan (Sac-TMT)					
Trofuse-009	NCT06305754	*Sacituzumab Tirumotecan*	Arm A: Sac-TMT 4 mg/kg q2w vs. Arm B: Pemetrexed 500 mg/m^2^ + Carboplatin AUC 5 q3w ×4 → Pemetrexed 500 mg/m^2^ q3w	III	Pretreated
Trofuse-004	NCT06074588	*Sacituzumab Tirumotecan*	Arm A: Sac-TMT 4 mg/kg on Days 1, 15, 29 of every 6-week cycle vs. Arm B: Docetaxel 75 mg/m^2^ q3w or Pemetrexed 500 mg/m^2^ q3w on Days 1, 22 of every 6-week cycle	III	Pretreated

Abbreviations: ADC, antibody-drug conjugate; AUC, area under the curve; CT, chemotherapy; Dato-DXd, datopotamab deruxtecan; EGFR, epidermal growth factor receptor; NCT, national clinical trial; q2w, every 2 weeks; q3w, every 3 weeks; Sac-TMT, sacituzumab tirumotecan.

## Data Availability

No new data were created or analyzed in this study. Data sharing is not applicable to this article.
